# Machine learning-based models for prediction of the risk of stroke in coronary artery disease patients receiving coronary revascularization

**DOI:** 10.1371/journal.pone.0296402

**Published:** 2024-02-08

**Authors:** Lulu Lin, Li Ding, Zhongguo Fu, Lijiao Zhang

**Affiliations:** 1 Department of Neurology, The Second Hospital of Dalian Medical University, Dalian, Liaoning, China; 2 Department of Neurology, Shenyang First People’s Hospital, Shenyang, Liaoning, China; 3 Department of Cardiology, The Second Hospital of Dalian Medical University, Dalian, Liaoning, China; Khalifa University, UNITED ARAB EMIRATES

## Abstract

**Background:**

To construct several prediction models for the risk of stroke in coronary artery disease (CAD) patients receiving coronary revascularization based on machine learning methods.

**Methods:**

In total, 5757 CAD patients receiving coronary revascularization admitted to ICU in Medical Information Mart for Intensive Care IV (MIMIC-IV) were included in this cohort study. All the data were randomly split into the training set (n = 4029) and testing set (n = 1728) at 7:3. Pearson correlation analysis and least absolute shrinkage and selection operator (LASSO) regression model were applied for feature screening. Variables with Pearson correlation coefficient<9 were included, and the regression coefficients were set to 0. Features more closely related to the outcome were selected from the 10-fold cross-validation, and features with non-0 Coefficent were retained and included in the final model. The predictive values of the models were evaluated by sensitivity, specificity, area under the curve (AUC), accuracy, and 95% confidence interval (CI).

**Results:**

The Catboost model presented the best predictive performance with the AUC of 0.831 (95%CI: 0.811–0.851) in the training set, and 0.760 (95%CI: 0.722–0.798) in the testing set. The AUC of the logistic regression model was 0.789 (95%CI: 0.764–0.814) in the training set and 0.731 (95%CI: 0.686–0.776) in the testing set. The results of Delong test revealed that the predictive value of the Catboost model was significantly higher than the logistic regression model (*P*<0.05). Charlson Comorbidity Index (CCI) was the most important variable associated with the risk of stroke in CAD patients receiving coronary revascularization.

**Conclusion:**

The Catboost model was the optimal model for predicting the risk of stroke in CAD patients receiving coronary revascularization, which might provide a tool to quickly identify CAD patients who were at high risk of postoperative stroke.

## Introduction

Coronary artery disease (CAD) is the most common cardiovascular diseases wherein atherosclerosis occurs in one or more of the coronary arteries [1]. CAD was reported to be one of the major causes of mortality in both the developed and developing countries [2]. Currently, percutaneous coronary intervention (PCI) and coronary artery bypass grafting (CABG) are common coronary revascularization procedures [3]. With the development and application of drug-eluting stents and minimally invasive surgery, the prognosis of patients undergoing PCI or CABG was improved, but some patients still have postoperative adverse cardiovascular events, which result in worse prognosis [4]. Stroke is a cerebrovascular disorder which is the second leading cause of mortality and morbidity worldwide [5]. Stroke is a prevalent complication among surgical and ICU patients, with postoperative stroke incidence in cardiac surgery patients ranging from 0.8% to 9% [6]. Previous evidence suggested that the occurrence of stroke occurrence was associated with a significantly elevated risk of mortality in patients undergoing PCI or CABG procedures [7, 8]. Constructing predictive models to accurately identify patients receiving coronary revascularization who were at high risk of stroke is of great significance.

Recently, machine learning methods are gradually applied to the construction of clinical models in order to improve the accuracy of clinical diagnosis or prediction of diseases [9]. Machine learning method was widely applied in predicting poor prognosis after heart surgery and the risk of postoperative stroke, which presented better performance than traditional risk models such as logistic regression [10–12]. However, no studies have reported the use of machine learning to predict the risk of postoperative stroke in patients undergoing coronary revascularization.

This study intended to construct several prediction models for the risk of stroke in CAD patients who underwent coronary revascularization based on machine learning methods. The optimal prediction model was identified and the predictive value was compared with traditional logistic regression model.

## Methods

### Study design and population

In this cohort study, the records of 6289 CAD patients receiving coronary revascularization were obtained in Medical Information Mart for Intensive Care IV (MIMIC-IV). MIMIC-IV builds upon the success of MIMIC-III and incorporates numerous enhancements from 2008 to 2019. MIMIC-IV is a relational database that encompasses authentic hospitalizations of patients admitted to a tertiary academic medical center located in Boston, MA, USA. Each patient’s length of stay, laboratory tests, medication treatment, vital signs and other comprehensive information during their ICU stay were recorded [13]. Patients with age < 18 years old and those with the length of ICU stay less than 24 h were excluded. Finally, 5757 participants were included. The requirement of ethical approval for this was waived by the Institutional Review Board of The second hospital of Dalian medical university, because the data was accessed from MIMIC-IV (a publicly available database). The need for written informed consent was waived by the Institutional Review Board of The second hospital of Dalian medical university due to retrospective nature of the study.

### Potential predictors

Age (years), gender (female or male), ethnicity (White, Black, others or unknown), insurance (Medicaid, Medicare or others), marital status (married, or no married), first care unit [Cardiac Care Unit (CCU), cardiovascular intensive care unit (CVICU) or others), family history of stroke (yes or no), personal history of stroke (yes or no), treatments (CABG and PCI, CABG alone or PCI alone), thrombolysis (yes or no), antiplatelet (yes or no), beta-blockers (yes or no), calcium channel blockers (yes or no), ventilation (yes or no), vasopressors (yes or no), Glasgow Coma Scale (GCS) score, Charlson Comorbidity Index (CCI), weight (kg), heart rate (bpm), systolic blood pressure (SBP) (mmHg), diastolic blood pressure (DBP) (mmHg), respiratory rate (bpm), temperature (°C), oxygen saturation (SpO_2_) (%), white blood cell (WBC) (K/uL), platelet (K/uL), hemoglobin (g/dL), red blood cell distribution width (RDW) (%), hematocrit (%), creatinine (mg/dL), international normalized ratio (INR), prothrombin time (PT) (seconds), partial thromboplastin time (PTT) (seconds), blood urea nitrogen (BUN) (mg/dL), glucose (mg/dL), calcium (mmol/L), sodium (mEq/L), chloride (mEq/L), bicarbonate (mEq/L), and lactate (mmol/L) were potential predictors. All these data were collected within 24 hours of admission to ICU.

### Construction of the prediction models

Logistic regression model is a classification algorithm evolved from linear regression, and belongs to a Sigmoid function normalization model of generalized linear regression model, which is commonly used to solve binary classification problems and has strong explanatory ability [14].

Support vector machine (SVM) is a classification algorithm, and it can also be classified. Different models can be made according to different input data. If the input label is classified value, SVC() is used for classification. This algorithm improves the generalization ability of learning machine by seeking the minimum structural risk, and minimizes the empirical risk and confidence range. Its basic model is defined as the linear classifier with the largest interval on the feature space, that is, the learning strategy of support vector machine is to maximize the interval, and it can be converted into the solution of a convex quadratic programming problem [15].

Random forest is an extended variant of Bagging ensemble learning. On the basis of constructing Bagging ensemble with decision tree as a base learner, it further adds the selection of random attributes to the training process of decision tree. For each node of the base decision tree, a subset containing k attributes is randomly selected from the candidate attribute set of the node. Then an optimal attribute is selected for division. The method of prediction stage of this algorithm is Bagging strategy. The classification model uses voting method to determine the final result, and the regression model uses mean method to determine the final result [16].

Extreme Gradient Boosting (XGBoost) is an efficient gradient lifting decision tree algorithm, which is improved on the basis of the original Gradient Boosting Decision Tree. As a forward addition model, its core is to adopt the Boosting thought, which integrates multiple weak learners into a strong learner by a certain method, that is, multiple trees make decisions together, and the result of each tree is the difference between the target value and the predicted result of all the previous trees, adding up all the results to get the final result. In this way, the effect of the whole model is improved [17].

Adaptive Boosting (Adaboost) is an iterative algorithm to train different classifiers for the same training set, and then set these weak classifiers together to form a stronger final classifier. The set strategy is to increase the weight of the samples that were classified wrong by the previous round of classifiers. Reducing the weight of samples with correct classification will get more attention from the following classifiers, and then weaker classifiers can be generated. By combining these weak classifiers with majority weighted voting, the classifier with small error rate is increased, and the classifier with large error rate is reduced, so that it plays a less role in voting [18].

Naive bayes is one of the most widely used classification algorithms. It is a classifier method based on Bayesian definition and independent assumption of feature conditions. Naive Bayes algorithm is based on Bayesian principle and uses the knowledge of probability statistics to classify sample data sets. It is characterized by the combination of prior probability and posterior probability, which avoids the subjective bias of using only prior probability, and also avoids the overfitting phenomenon of using sample information alone [19].

K-nearest neighbor (KNN) is one of the most basic and simplest algorithms in the machine learning algorithm model. It can be used for classification and regression by measuring the distance between different eigenvalues. The working principle is to use the training data to partition the eigenvector space and take the partition result as the final algorithm model.

Categorical boosting (Catboost) is a kind of gradient boosting algorithm library that can handle categorical features well. It has made some improvements on the basis of the original Gradient Boosting Decision Tree. Specifically, the algorithm has two characteristics of adaptive learning rate and categorical feature processing, which can help the algorithm better control the contribution of the weak learner in each iteration. In addition, the algorithm can deal with categorical features efficiently and reasonably, so it can deal with the influence of categorical features better [20].

### Outcome variable

Postoperative stroke was the outcome, which was screened according to the ICD diagnosis codes, ICD-9 (430–436, and 997020, and ICD-10 three I60-I66, I9782, I97820.

### Statistical analysis

Mean ± SD was used to describe the distribution of measurement data subject to normal distribution, and t-test was used to compare the difference between groups. Median and quartiles were used to describe the distribution of measurement data that did not follow normal distribution, and Wilcoxon rank sum test was used to compare the difference between groups. The enumeration data were expressed as number and percentage of cases [n (%)], and the Chi-square test or Fisher’s exact probability were used to compare the differences between the groups. Missing values <20% were dealt by Random forest interpolation, and ≥20% were deleted (Table 1). Sensitivity analysis were performed before and after interpolation (Table 2). All the data were randomly split into the training set (n = 4029) and testing set (n = 1728) at 7:3 with the random seed if 42 [21]. Pearson correlation analysis and least absolute shrinkage and selection operator (LASSO) regression model were applied for feature screening. Variables with Pearson correlation coefficient<9 were included, and the regression coefficients were set to 0. Features more closely related to the outcome were selected from the 10-fold cross-validation, and features with non-0 Coefficient were retained and included in the final model. Eight prediction models were constructed, and the parameter settings were shown in Table 3. The predictive values of the models were evaluated by sensitivity, specificity, area under the curve (AUC), accuracy, and 95% confidence interval (CI). The confidence level alpha = 0.05. Missing value interpolation, training set and testing set split, data modeling and result visualization were completed using Python 3.9.12. Sensitivity analysis and difference comparison were performed by SAS 9.4 (SAS Institute Inc., Cary, NC, USA).

**Table 1:** The number and percentage of missing values.

Variables	n	%	Total samples (n)	Manipulation
GCS	6	0.10%	5751	Random forest interpolation
SPO_2_	6	0.10%	5751	Random forest interpolation
Systolic	7	0.12%	5750	Random forest interpolation
Diastolic	7	0.12%	5750	Random forest interpolation
Platelet	7	0.12%	5750	Random forest interpolation
Heart rate	8	0.14%	5749	Random forest interpolation
Chloride	8	0.14%	5749	Random forest interpolation
Sodium	9	0.16%	5748	Random forest interpolation
BUN	11	0.19%	5746	Random forest interpolation
Creatinine	11	0.19%	5746	Random forest interpolation
Hematocrit	12	0.21%	5745	Random forest interpolation
Bicarbonate	15	0.26%	5742	Random forest interpolation
Glucose	16	0.28%	5741	Random forest interpolation
RDW	18	0.31%	5739	Random forest interpolation
Hemoglobin	18	0.31%	5739	Random forest interpolation
WBC	18	0.31%	5739	Random forest interpolation
Calcium	35	0.61%	5722	Random forest interpolation
Weight	135	2.34%	5622	Random forest interpolation
PTT	147	2.55%	5610	Random forest interpolation
INR	149	2.59%	5608	Random forest interpolation
PT	150	2.61%	5607	Random forest interpolation
Marital status	358	6.22%	5399	Random forest interpolation
Temperature	685	11.90%	5072	Random forest interpolation
Lactate	734	12.75%	5023	Random forest interpolation
Respiratory rate	783	13.60%	4974	Random forest interpolation
Lymphocytes	2514	43.67%	3243	Deleting
Neutrophil	2514	43.67%	3243	Deleting
ALT	4976	86.43%	781	Deleting
Bilirubin total	4977	86.45%	780	Deleting
AST	4983	86.56%	774	Deleting
ALP	5012	87.06%	745	Deleting
Albumin	5377	93.40%	380	Deleting
Cholesterol	5460	94.84%	297	Deleting
Cholesterol-HDL	5464	94.91%	293	Deleting
Triglycerides	5466	94.95%	291	Deleting
Cholesterol-LDL	5467	94.96%	290	Deleting
Height	5498	95.50%	259	Deleting
Ntprobnp	5628	97.76%	129	Deleting
GGT	5753	99.93%	4	Deleting
Troponin-I	5757	100.00%	0	Deleting

GCS: Glasgow Coma Scale, SPO_2_: oxygen saturation, BUN, blood urea nitrogen, RDW: red blood cell distribution width, WBC: white blood cell, PTT partial thromboplastin time, INR: international normalized ratio, PT prothrombin time, HDL: high density lipoprotein

**Table 2:** Sensitivity analysis of data before and after manipulation.

Variables	After manipulation (n = 5757)	Before manipulation (n = 5757)	Statistics	*P*
Platelet, K/uL, Mean ± SD	159.42 ± 51.43	157.92 ± 51.44	t = 1.55	0.121
Calcium, mmol/L, M (Q_1_, Q_3_)	1.20 (1.11, 1.35)	1.20 (1.11, 1.35)	Z = -0.413	0.679
SPO_2_, %, Mean ± SD	98.81 ± 2.63	98.82 ± 2.63	t = -0.02	0.985
RDW, %, Mean ± SD	13.60 ± 0.99	13.57 ± 1.00	t = 1.44	0.151
BUN, mg/dL, M (Q_1_, Q_3_)	16.00 (13.00, 21.00)	16.00 (13.00, 21.00)	Z = -0.011	0.991
Glucose, mg/dL, Mean ± SD	139.92 ± 33.61	139.41 ± 34.17	t = 0.79	0.427
Marital status, n (%)			χ^2^ = 1.459	0.227
Married	3854 (66.94)	3556 (65.86)		
No married	1903 (33.06)	1843 (34.14)		
Creatinine, mg/dL, M (Q_1_, Q_3_)	0.90 (0.70, 1.10)	0.90 (0.70, 1.10)	Z = 0.025	0.980
Lactate, mmol/L, M (Q_1_, Q_3_)	2.00 (1.60, 2.60)	2.10 (1.50, 2.70)	Z = 1.470	0.141
Weight, kg, Mean ± SD	84.67 ± 16.98	84.51 ± 17.21	t = 0.49	0.622
Hemoglobin, g/dL, Mean ± SD	10.27 ± 2.27	10.27 ± 2.27	t = 0.06	0.955
Temperature, °C, Mean ± SD	36.35 ± 0.47	36.36 ± 0.51	t = -1.75	0.080
INR, ratio, Mean ± SD	1.38 ± 0.19	1.38 ± 0.20	t = 0.50	0.620
Chloride, mEq/L, Mean ± SD	105.84 ± 3.60	105.91 ± 3.58	t = -0.97	0.334
PT, seconds, Mean ± SD	15.15 ± 2.02	15.12 ± 2.05	t = 0.89	0.375
PTT, seconds, Mean ± SD	37.23 ± 21.82	36.66 ± 21.72	t = 1.39	0.165
Hematocrit, %, Mean ± SD	30.77 ± 6.67	30.74 ± 6.66	t = 0.25	0.800
Heart rate, bpm, Mean ± SD	80.04 ± 9.38	80.00 ± 9.52	t = 0.19	0.848
SBP, mmHg, Mean ± SD	113.69 ± 16.67	113.49 ± 16.70	t = 0.65	0.516
DBP, mmHg, Mean±SD	59.74 ± 10.73	59.43 ± 10.66	t = 1.52	0.128
Respiratory rate, bpm, Mean ± SD	15.21 ± 2.07	15.18 ± 2.34	t = 0.61	0.542
Bicarbonate, mEq/L, Mean ± SD	23.18 ± 2.17	23.19 ± 2.20	t = -0.27	0.790
Sodium, mEq/L, Mean ± SD	135.76 ± 2.83	135.76 ± 2.84	t = -0.10	0.920
WBC, K/uL, M (Q_1_, Q_3_)	12.20 (9.20, 15.50)	12.10 (9.20, 15.50)	Z = -0.730	0.466
GCS, score, Mean ± SD	13.38 ± 3.54	13.38 ± 3.54	t = 0.02	0.985

SD: standard deviation, M: median, Q1: 1st quartile, Q3: 3st quartile, SPO_2_: oxygen saturation, RDW: red blood cell distribution width, BUN, blood urea nitrogen, INR: international normalized ratio, PT prothrombin time, PTT partial thromboplastin time, SBP: systolic blood pressure, DBP: diastolic blood pressure, WBC: white blood cell, GCS: Glasgow Coma Scale

**Table 3:** Parameter settings for 8 machine learning models.

Model	Logistic regression	SVM	Random forest	XGBoost	Adaboost	Naive bayes	KNN	Catboost
Parameter 1	C = 1	kernel = ’rbf’	n_jobs = -1	learning_rate = 0.001	learning_rate = 0.001	alpha = 10	n_neighbors = 13	objective = ‘ CrossEntropy’
Parameter 2	max_iter = 1000	probability = True	oob_score = True	max_depth = 4	n_estimators = 178		weights = ‘ uniform’	colsample_bylevel = 0.503349
Parameter 3	n_jobs = -1	C = 0.732536	class_weight = ’balanced’	n_estimators = 494	random_state = 151		leaf_size = 35	depth = 4
Parameter 4	random_state = 151	gamma = 0.011125	n_estimators = 300	min_child_weight = 6				l2_leaf_reg = 1
Parameter 5	solver = ’newton-cg’	random_state = 151	max_depth = 5	subsample = 0.218971				boosting_type = ‘Ordered’
Parameter 6	penalty = ’l2’		random_state = 151	reg_lambda = 1				bootstrap_type = ‘MVS’
Parameter 7				Seed = 151				random_state = 151
Parameter 8								used_ram_limit = ‘3gb’
Parameter 9								learning_rate = 0.001

SVM: support vector machine, XGBoost: Extreme Gradient Boosting, Adaboost: Adaptive Boosting, KNN: K-nearest neighbor, Catboost: Categorical boosting

## Results

### Comparisons of the characteristics of participants with and without postoperative stroke in the training set

**Fig 1:**
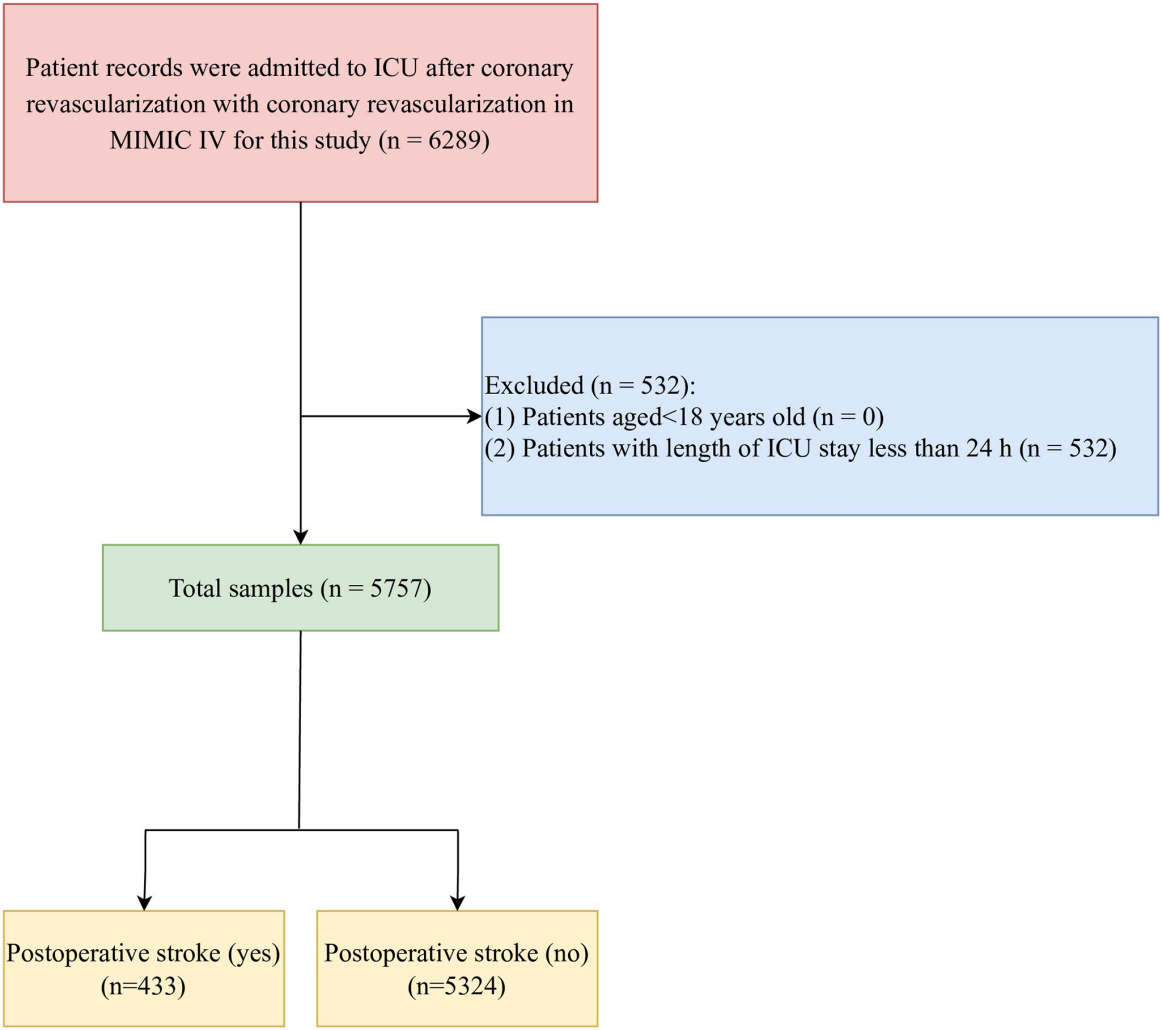
The screen process of the participants.

A total of 6289 CAD patients undergoing coronary revascularization were identified in MIMIC-IV. Among them, patients with the length of ICU stay less than 24 h were excluded (n = 532). Finally, 5757 participants were included. All patients were divided into the postoperative stroke group (n = 433) and postoperative non-stroke group according whether postoperative stroke occurred. The screen process of the participants was exhibited in Fig 1.

The percentage of patients with personal history of stroke in those with postoperative stroke was higher those without (15.86% vs 5.35%; *P*<0.001). The mean INR in patient with postoperative stroke was higher those without (1.41 vs 1.37; *P* = 0.001). The median length of stay in patents with postoperative stroke was higher those without (2.31 days vs 1.83 days; *P*<0.001). More detailed information was observed in Table 4.

**Table 4:** Comparisons of the characteristics of participants with and without postoperative stroke in the training set.

		Postoperative stroke			
Variables	Total (n = 4029)	No (n = 3720)	yes (n = 309)	Statistics	*P*
Age, years, Mean ± SD	67.77 ± 10.96	67.45 ± 11.00	71.54 ± 9.85	t = -6.94	<0.001
Gender, n (%)				χ^2^ = 8.998	0.003
Female	1005 (24.94)	906 (24.35)	99 (32.04)		
Male	3024 (75.06)	2814 (75.65)	210 (67.96)		
Ethnicity, n (%)				χ^2^ = 4.675	0.197
White	2881 (71.51)	2650 (71.24)	231 (74.76)		
Black	162 (4.02)	155 (4.17)	7 (2.27)		
Others	483 (11.99)	453 (12.18)	30 (9.71)		
Unknown	503 (12.48)	462 (12.42)	41 (13.27)		
Insurance, n (%)				χ^2^ = 13.545	0.001
Medicaid	130 (3.23)	120 (3.23)	10 (3.24)		
Medicare	1819 (45.15)	1649 (44.33)	170 (55.02)		
Others	2080 (51.63)	1951 (52.45)	129 (41.75)		
Marital status, n (%)				χ^2^ = 5.110	0.024
Married	2686 (66.67)	2498 (67.15)	188 (60.84)		
No married	1343 (33.33)	1222 (32.85)	121 (39.16)		
First care unit, n (%)				χ^2^ = 4.078	0.130
CCU	595 (14.77)	561 (15.08)	34 (11.00)		
CVICU	3388 (84.09)	3117 (83.79)	271 (87.70)		
Others	46 (1.14)	42 (1.13)	4 (1.29)		
Family history of stroke, n (%)				-	0.653
No	4010 (99.53)	3703 (99.54)	307 (99.35)		
Yes	19 (0.47)	17 (0.46)	2 (0.65)		
Personal history of stroke, n (%)				χ^2^ = 54.537	<0.001
No	3781 (93.84)	3521 (94.65)	260 (84.14)		
Yes	248 (6.16)	199 (5.35)	49 (15.86)		
Treatments, n (%)				χ^2^ = 4.197	0.123
CABG and PCI	22 (0.55)	21 (0.56)	1 (0.32)		
CABG alone	3316 (82.30)	3049 (81.96)	267 (86.41)		
PCI alone	691 (17.15)	650 (17.47)	41 (13.27)		
Thrombolysis, n (%)				-	1.000
No	4027 (99.95)	3718 (99.95)	309 (100.00)		
Yes	2 (0.05)	2 (0.05)	0 (0.00)		
Antiplatelet, n (%)				-	0.321
No	3970 (98.54)	3663 (98.47)	307 (99.35)		
Yes	59 (1.46)	57 (1.53)	2 (0.65)		
Beta blockers, n (%)				χ^2^ = 18.061	<0.001
No	2933 (72.80)	2740 (73.66)	193 (62.46)		
Yes	1096 (27.20)	980 (26.34)	116 (37.54)		
Calcium channel blockers, n (%)				χ^2^ = 22.544	<0.001
No	3616 (89.75)	3363 (90.40)	253 (81.88)		
Yes	413 (10.25)	357 (9.60)	56 (18.12)		
Ventilation, n (%)				χ^2^ = 4.717	0.030
No	176 (4.37)	170 (4.57)	6 (1.94)		
Yes	3853 (95.63)	3550 (95.43)	303 (98.06)		
Vasopressors, n (%)				χ^2^ = 8.987	0.003
No	1060 (26.31)	1001 (26.91)	59 (19.09)		
Yes	2969 (73.69)	2719 (73.09)	250 (80.91)		
GCS, score, Mean ± SD	13.36 ± 3.55	13.40 ± 3.52	12.89 ± 3.90	t = 2.21	0.028
CCI, score, M (Q_1_, Q_3_)	2.00 (1.00, 3.00)	2.00 (1.00, 3.00)	3.00 (2.00, 5.00)	Z = 14.468	<0.001
Weight, kg, Mean ± SD	84.60 ± 16.96	84.92 ± 16.98	80.81 ± 16.34	t = 4.10	<0.001
Heart rate, bpm, Mean ± SD	80.03 ± 9.40	80.07 ± 9.44	79.58 ± 8.94	t = 0.88	0.379
SBP, mmHg, Mean ± SD	113.64 ± 16.80	113.68 ± 16.76	113.17 ± 17.33	t = 0.51	0.610
DBP, mmHg, Mean ± SD	59.69 ± 10.76	59.92 ± 10.67	56.98 ± 11.39	t = 4.63	<0.001
Respiratory rate, bpm, Mean ± SD	15.21 ± 2.08	15.21 ± 2.09	15.20 ± 1.98	t = 0.11	0.916
Temperature, °C, Mean ± SD	36.34 ± 0.47	36.34 ± 0.47	36.31 ± 0.49	t = 1.40	0.162
SPO_2_, %, Mean ± SD	98.79 ± 2.74	98.78 ± 2.73	98.88 ± 2.83	t = -0.59	0.556
WBC, K/uL, M (Q_1_, Q_3_)	12.20 (9.30, 15.60)	12.30 (9.30, 15.60)	11.70 (8.70, 15.10)	Z = -1.343	0.179
Platelet, K/uL, M (Q_1_, Q_3_)	152.20 (121.00, 192.00)	153.00 (121.00, 192.00)	144.00 (118.00, 186.00)	Z = -1.714	0.086
Hemoglobin, g/dL, Mean ± SD	10.27 ± 2.28	10.33 ± 2.28	9.64 ± 2.24	t = 5.13	<0.001
RDW, %, Mean ± SD	13.60 ± 1.00	13.57 ± 1.00	13.86 ± 0.95	t = -4.79	<0.001
Hematocrit, %, Mean ± SD	30.77 ± 6.72	30.94 ± 6.71	28.79 ± 6.58	t = 5.40	<0.001
Creatinine, mg/dL, M (Q_1_, Q_3_)	0.90 (0.70, 1.10)	0.90 (0.70, 1.10)	0.90 (0.70, 1.10)	Z = -0.588	0.557
INR, ratio, Mean ± SD	1.38 ± 0.19	1.37 ± 0.19	1.41 ± 0.21	t = -3.24	0.001
PT, seconds, Mean ± SD	15.14 ± 2.00	15.11 ± 1.99	15.51 ± 2.11	t = -3.38	<0.001
PTT, seconds, Mean ± SD	37.36 ± 22.21	37.45 ± 22.57	36.28 ± 17.30	t = 1.12	0.266
BUN, mg/dL, M (Q_1_, Q_3_)	16.00 (13.00, 21.00)	16.00 (13.00, 21.00)	17.00 (14.00, 23.00)	Z = 1.946	0.052
Glucose, mg/dL, Mean ± SD	140.04 ± 33.37	140.03 ± 33.27	140.20 ± 34.62	t = -0.09	0.932
Calcium, mmol/L, M (Q_1_, Q_3_)	1.20 (1.11, 1.35)	1.20 (1.12, 1.36)	1.18 (1.11, 1.33)	Z = -1.864	0.062
Sodium, mEq/L, Mean ± SD	135.80 ± 2.85	135.82 ± 2.84	135.58 ± 2.90	t = 1.43	0.153
Chloride, mEq, Mean ± SD	105.86 ± 3.63	105.84 ± 3.63	106.09 ± 3.71	t = -1.16	0.245
Bicarbonate, mEq/L, Mean ± SD	23.20 ± 2.17	23.22 ± 2.17	22.98 ± 2.17	t = 1.83	0.068
Lactate, mmol/L, M (Q_1_, Q_3_)	2.01 (1.60, 2.60)	2.01 (1.60, 2.60)	2.01 (1.50, 2.80)	Z = 0.490	0.624
LOS, days, M (Q_1_, Q_3_)	1.92 (1.26, 3.11)	1.83 (1.25, 3.07)	2.31 (1.36, 3.64)	Z = 6.621	<0.001

SD: standard deviation, M: Median, Q_1_: 1st quartile, Q_3_: 3st quartile, CCU: Cardiac Care Unit, CVICU: cardiovascular intensive care unit, CABG: coronary artery bypass grafting, PCI: percutaneous coronary intervention, GCS: Glasgow Coma Scale, CCI: Charlson Comorbidity Index, SBP: systolic blood pressure, DBP: diastolic blood pressure, SPO_2_: oxygen saturation, WBC: white blood cell, RDW: red blood cell distribution width, INR: international normalized ratio (INR), PT prothrombin time, PTT partial thromboplastin time, BUN, blood urea nitrogen, LOS: length of stay

### Construction and the predictive values of the prediction models for the risk of stroke in CAD patients receiving coronary revascularization

**Fig 2:**
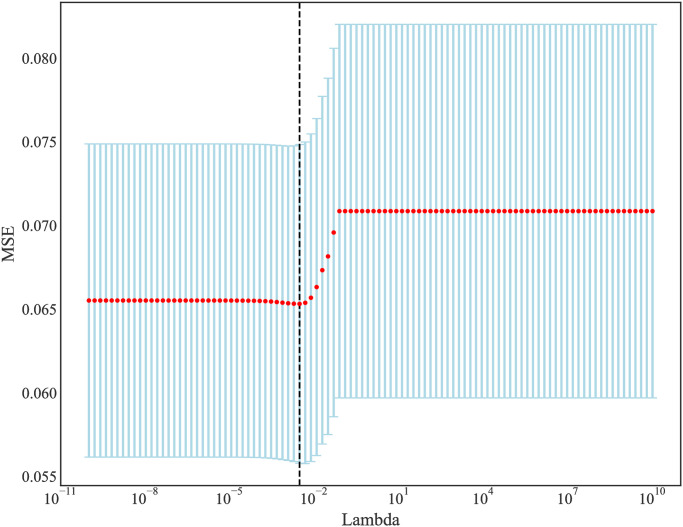
The changes of MSE with Lambda in the Lasso regression.

**Fig 3:**
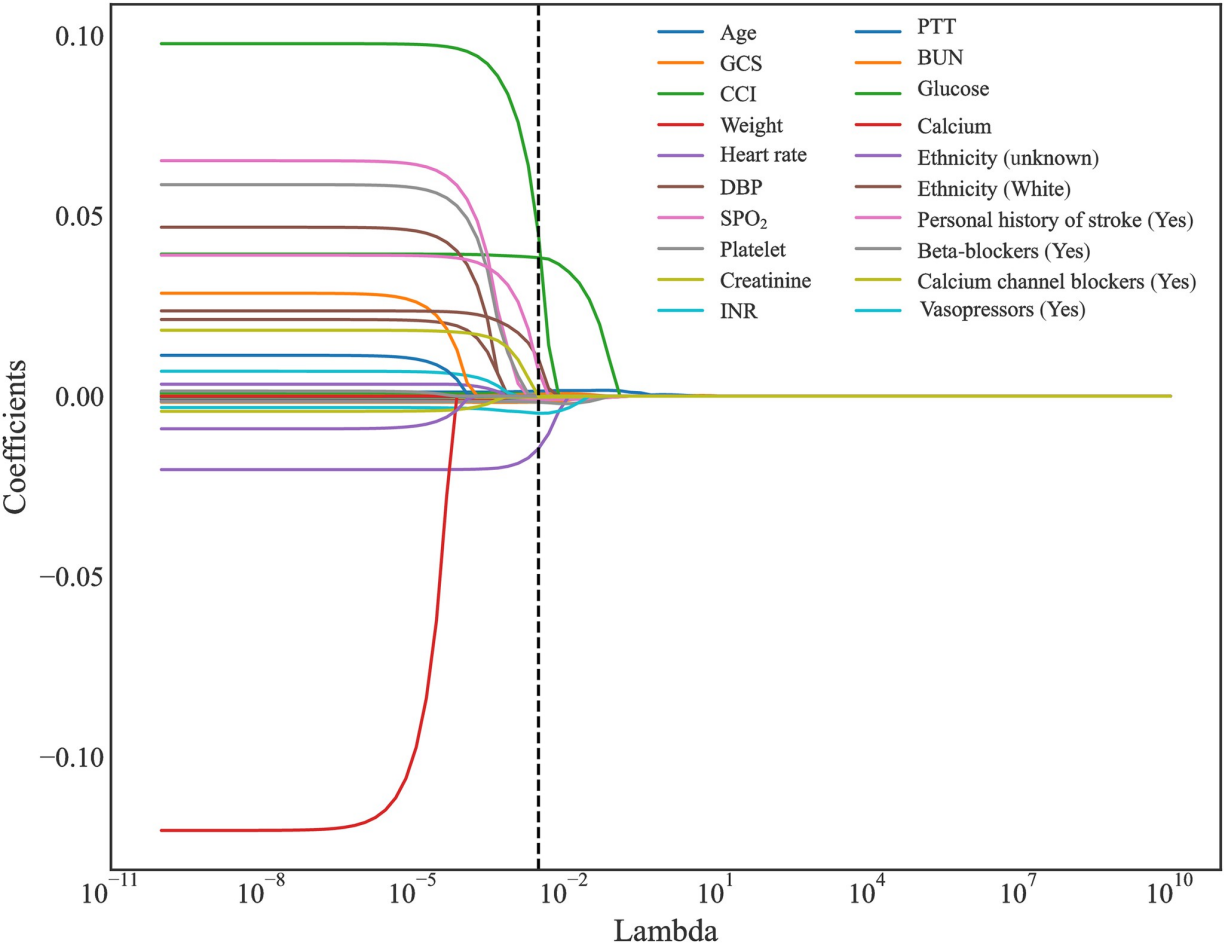
The changes of Coefficients with Lambda in the Lasso regression.

Initially, 40 features included, and 45 features were identified after one-hot encoding during the discretization of classification features. There were 39 variables with Pearson correlation coefficient<9. In order to ensure the stability and efficiency of features, valuable feature sets were selected from the 10-fold cross-validation results. As λ gradually expanded from 10^−10^ to 10^10^, the number of variables entering the model decreased. When λ was 0.002984, LASSO regression model showed the best prediction performance. Finally, 20 features with non-0 Coefficient were retained, which were age, GCS, CCI, weight, heart rate, DBP, SPO_2_, platelet, creatinine, INR, PTT, BUN, glucose, calcium, Ethnicity-unknown, Ethnicity-White, personal history of stroke-yes, beta-blockers-yes, calcium channel blockers-yes, and vasopressors-yes. Fig 2 presented the changes of mean-squared error (MSE), and Fig 3 showed the changes of Coefficients with Lambda in the Lasso regression.

### The predictive values of the prediction models for the risk of stroke in CAD patients undergoing coronary revascularization

**Fig 4:**
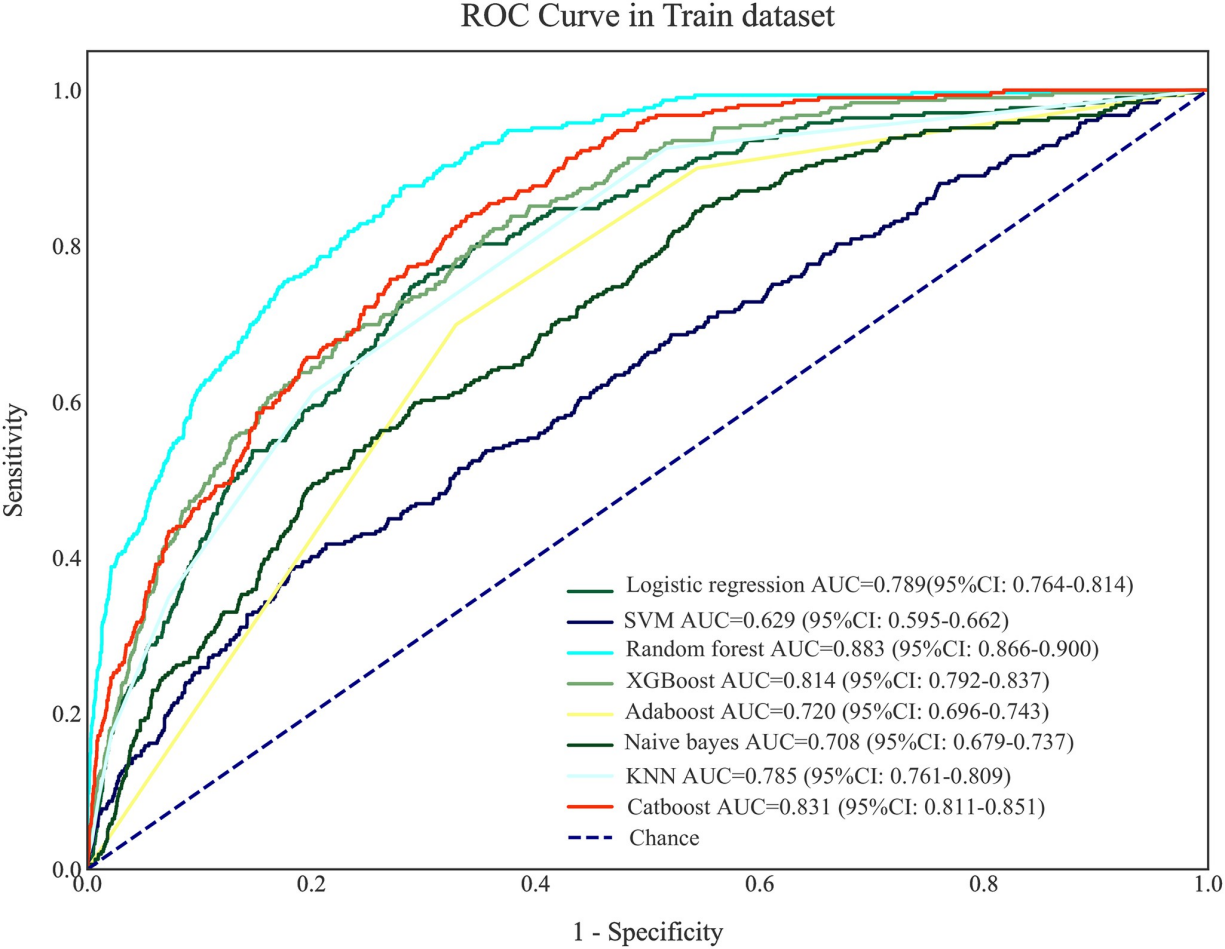
The ROC curves of machine learning models in the training set.

**Fig 5:**
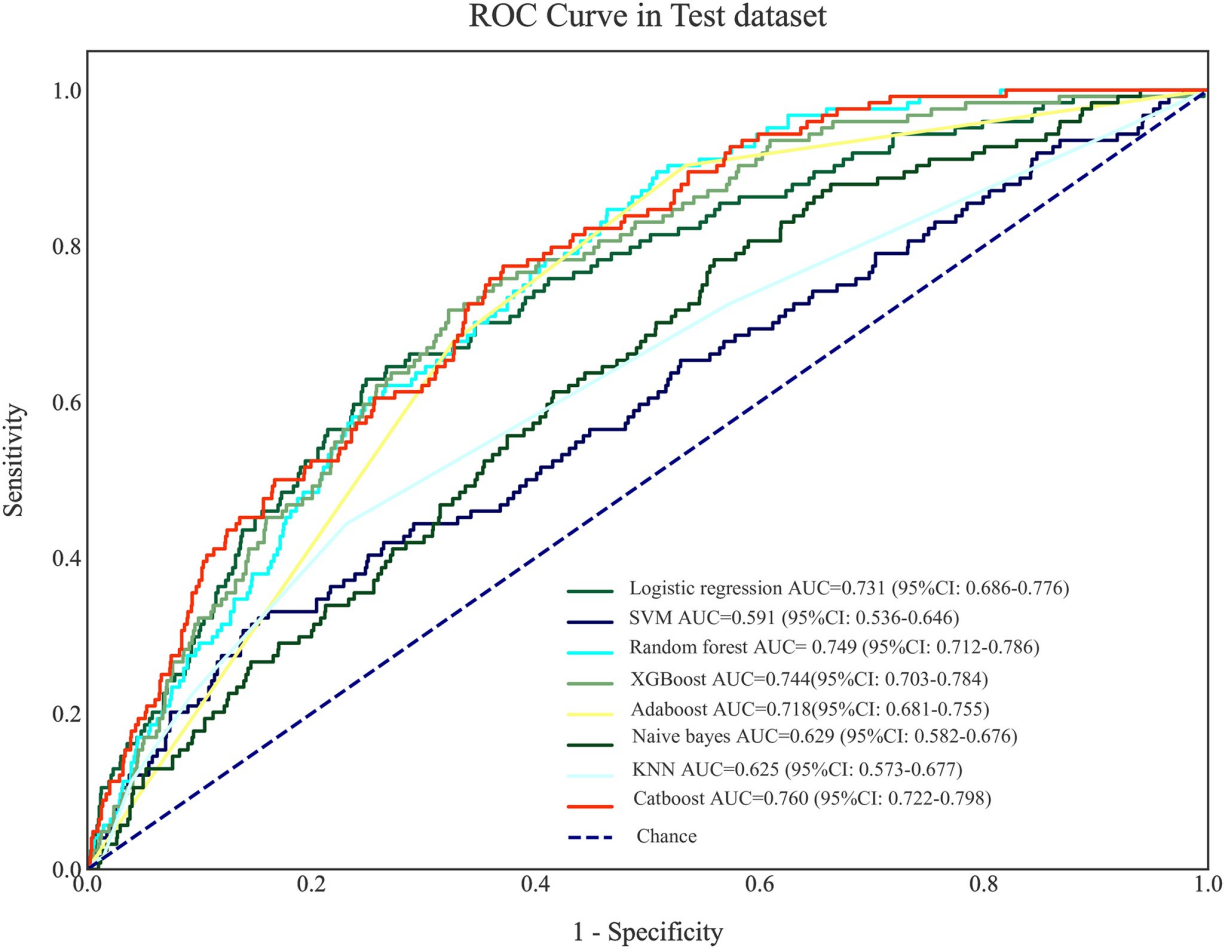
The ROC curves of machine learning models in the testing set.

The predictive values of prediction models for stroke in CAD patients undergoing coronary revascularization were presented in Table 5. The results delineated that Catboost model presented the best predictive performance with the AUC of 0.831 (95%CI: 0.811–0.851) in the training set, and 0.760 (95%CI: 0.722–0.798) in the testing set. The AUC of the logistic regression model was 0.789 (95%CI: 0.764–0.814) in the training set and 0.731 (95%CI: 0.686–0.776) in the testing set (Table 6). The results of Delong test revealed that the predictive value of the Catboost model was significantly higher than the logistic regression model (*P*<0.05). The ROC curves of machine learning models in the training set and testing set were respectively shown in Figs 4 and 5.

**Table 5:** The predictive values of different machine learning prediction models.

Model	Cut off	Sensitivity (95%CI)	Specificity (95%CI)	AUC (95%CI)	Accuracy (95%CI)
Logistic regression Training set	0.076	0.764 (0.716–0.811)	0.696 (0.681–0.711)	0.789 (0.764–0.814)	0.701 (0.687–0.715)
Logistic regression Testing set	0.076	0.669 (0.587–0.752)	0.671 (0.648–0.694)	0.731 (0.686–0.776)	0.671 (0.649–0.693)
SVM Training set	0.114	0.417 (0.362–0.472)	0.787 (0.774–0.800)	0.629 (0.595–0.662)	0.759 (0.746–0.772)
SVM Testing set	0.114	0.331 (0.248–0.413)	0.816 (0.797–0.835)	0.591 (0.536–0.646)	0.781 (0.762–0.801)
Random forest Training set	0.466	0.877 (0.840–0.914)	0.717 (0.703–0.732)	0.883 (0.866–0.900)	0.730 (0.716–0.743)
Random forest Testing set	0.466	0.621 (0.536–0.706)	0.734 (0.713–0.756)	0.749 (0.712–0.786)	0.726 (0.705–0.747)
XGBoost Training set	0.339	0.838 (0.797–0.879)	0.622 (0.606–0.638)	0.814 (0.792–0.837)	0.639 (0.624–0.653)
XGBoost Testing set	0.339	0.782 (0.710–0.855)	0.577 (0.553–0.601)	0.744 (0.703–0.784)	0.591 (0.568–0.615)
Adaboost Training set	0.148	0.699 (0.648–0.750)	0.671 (0.656–0.686)	0.720 (0.696–0.743)	0.673 (0.659–0.688)
Adaboost Testing set	0.148	0.677 (0.595–0.760)	0.673 (0.650–0.696)	0.718 (0.681–0.755)	0.674 (0.652–0.696)
Naïve bayes Training set	0.071	0.599 (0.544–0.653)	0.708 (0.694–0.723)	0.708 (0.679–0.737)	0.700 (0.686–0.714)
Naïve bayes Testing set	0.071	0.524 (0.436–0.612)	0.646 (0.622–0.669)	0.629 (0.582–0.676)	0.637 (0.614–0.660)
KNN Training set	0.154	0.612 (0.557–0.666)	0.798 (0.785–0.811)	0.785 (0.761–0.809)	0.784 (0.771–0.797)
KNN Testing set	0.154	0.226 (0.152–0.299)	0.906 (0.892–0.920)	0.625 (0.573–0.677)	0.857 (0.841–0.874)
Catboost Training set	0.173	0.838 (0.797–0.879)	0.662 (0.646–0.677)	0.831 (0.811–0.851)	0.675 (0.661–0.690)
Catboost Testing set	0.173	0.718 (0.639–0.797)	0.660 (0.637–0.683)	0.760 (0.722–0.798)	0.664 (0.642–0.687)

**Table 6:** Comparisons of the prediction performance of Catboost model and logistic regression model.

Dataset	Model	AUC (95%CI)	Statistics	*P*
Training set	Logistic regression	0.789 (0.764–0.814)	Ref	
	Catboost	0.831 (0.811–0.851)	Z = 6.36	<0.001
Testing set	Logistic regression	0.731 (0.686–0.776)	Ref	
	Catboost	0.760 (0.722–0.798)	Z = 3.12	0.002

**Fig 6:**
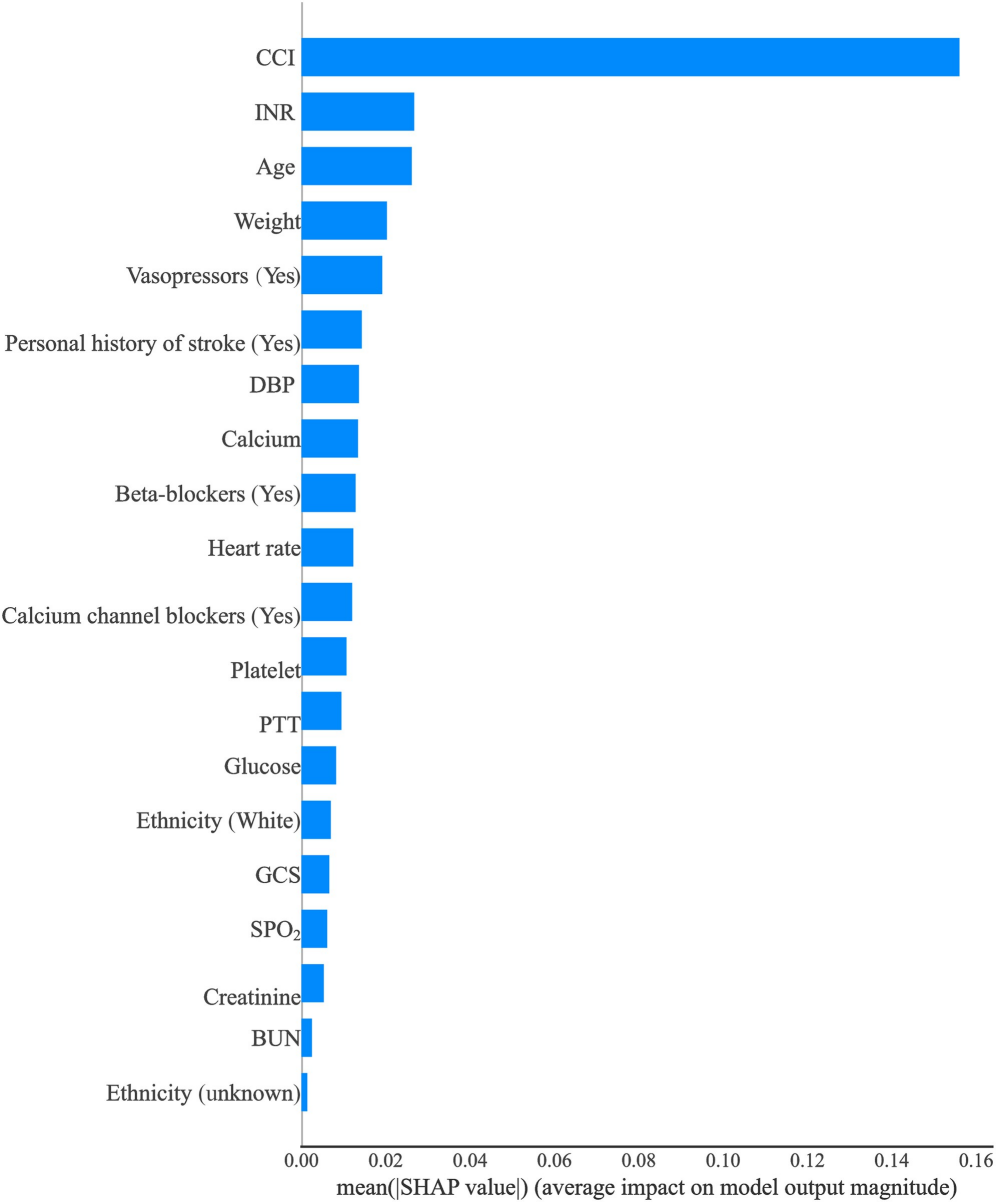
Absolut summary plot showing the importance of each feature in the Catboost model.

The importance of each feature in the Catboost model was displayed in Fig 6, which depicted that CCI was the most important variable associated with the risk of stroke in CAD patients undergoing coronary revascularization.

**Fig 7:**
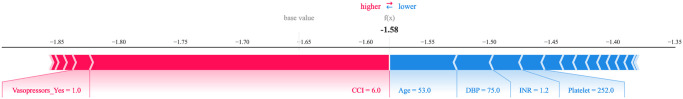
Summary plot for SHAP values of features in the Catboost model.

**Fig 8:**
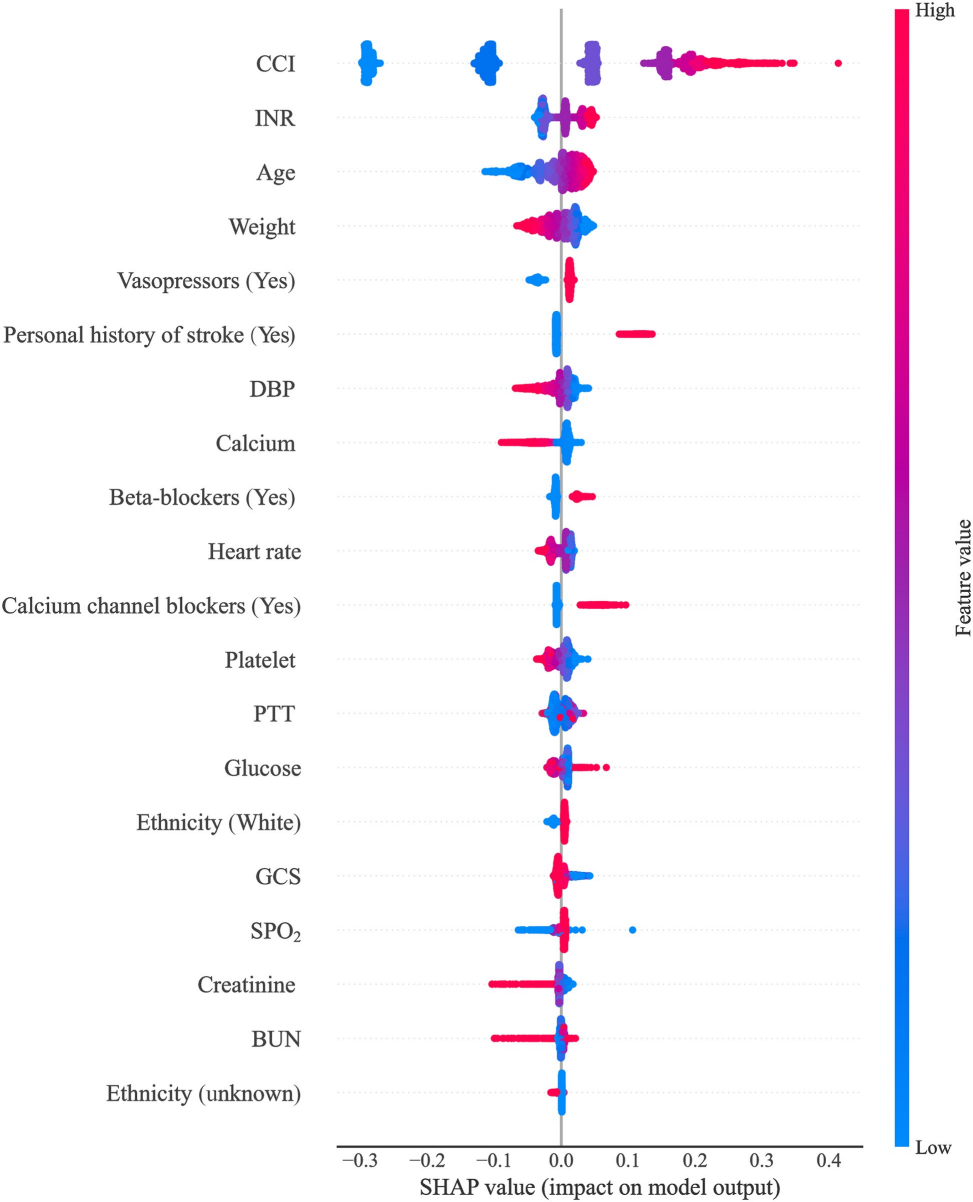
Force plot for SHAP values of features in the Catboost model.

**Fig 9:**
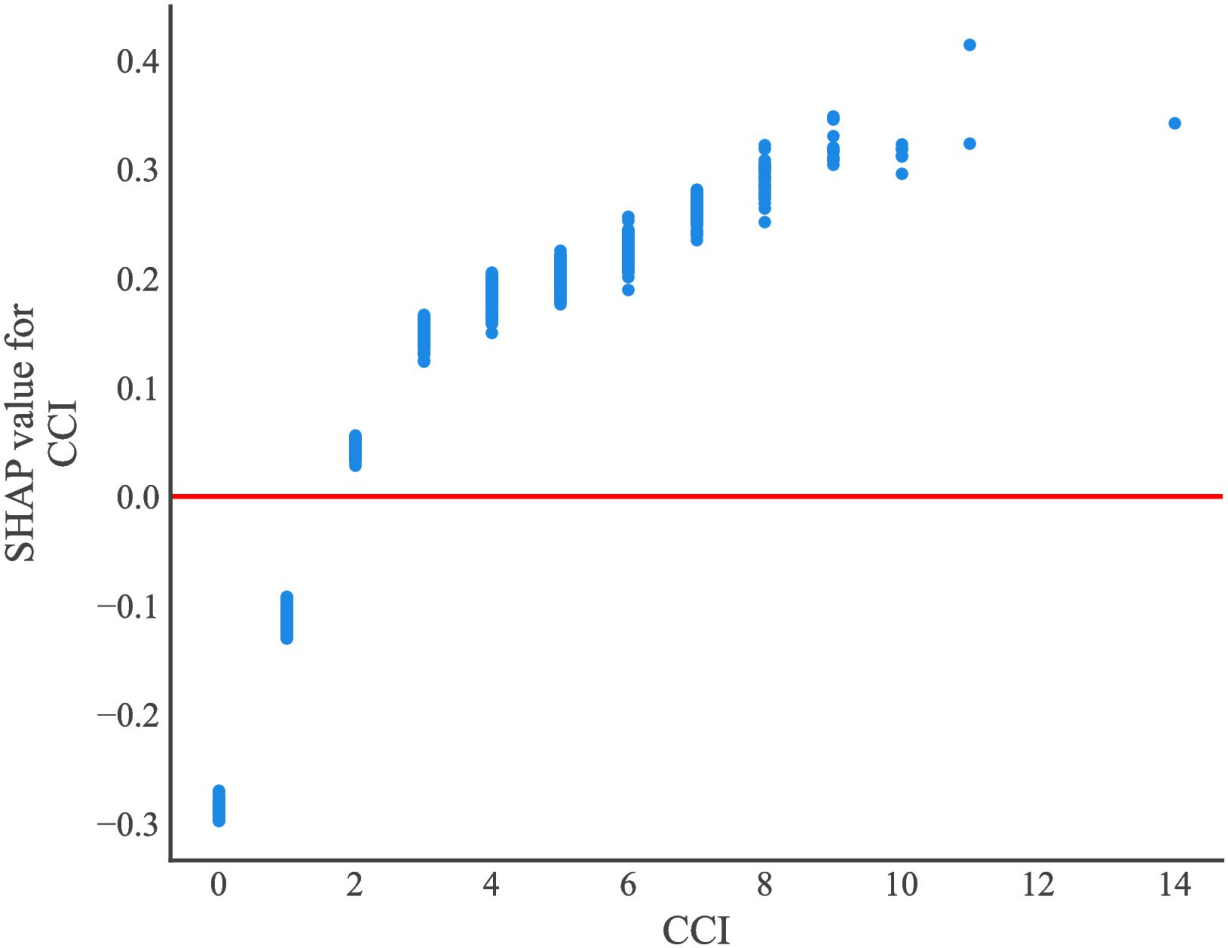
Dependence plot for SHAP values of CCI.

The SHapley Additive exPlanations (SHAP) values of features in the Catboost model were visualized in Fig 7, with SHAP values on the X-axis, features on the Y-axis, and each point representing a sample. The redder color indicates a stronger effect of the feature on the outcome, while the bluer color indicates a weaker effect. CCI was an important factor that exhibited a positive correlation with the risk of stroke in CAD patients following coronary revascularization. Creatinine levels were found to be associated with the risk of stroke, as indicated by blue dots primarily concentrated in areas where SHAP values exceeded 0, suggesting that lower creatinine levels were linked to higher stroke risk. Fig 8 depicted the SHAP value analysis of each sample in the Catboost model, where blue represents negative feature contribution and red indicates positive contribution. The length of an arrow signifies the degree of influence that a feature has on output, and its reduction or increase can be observed through the scale value on the X-axis. Base value refers to the average output of the model and training data, while the number below the arrow represents the actual eigenvalue of a single sample. Referring to a single sample, we found that CCI had the reddest summary plots, indicating that patients with a CCI score of 6 might be at risk for postoperative stroke. Age exhibited the longest blue bar, suggesting that patients aged 53 might experience reduced risk of postoperative stroke. The final SHAP output value of the model was -1.58. Fig 9 illustrated the impact of each feature in the Catboost prediction model on the model’s output. The x-axis represents features, while the y-axis represents SHAP values, and each dot denotes a sample. When a feature’s SHAP value exceeded 0, the risk of postoperative stroke was increased. CCI≥2 was associated with an increased risk of stroke.

## Discussion

The present study constructed several prediction models for the risk of stroke in CAD patients who received coronary revascularization based on machine learning methods. The results demonstrated that Catboost model was the optimal model for predicting the risk of stroke in CAD patients who received coronary revascularization. The AUC of Catboost model was 0.831 in the training set, and 0.760 in the testing set, which were higher than the logistic regression model. The findings might provide a novel and quick tool to identify CAD patients receiving coronary revascularization treatments who were at high risk of stroke, and offer timely interventions to prevent the poor prognosis.

Previously, several prediction models were constructed to predict the risk of cardiovascular events in CAD patients receiving coronary revascularization. Zhang et al. built a nomogram for predicting major adverse cardiovascular events after PCI in coronary heart disease patients with chronic kidney disease, and the AUC value of the model was 0.612 [22]. Another prediction model for predicting major adverse cardiac and cerebrovascular events among high-risk myocardial infarction patients undergoing primary PCI had an AUC of 0.883 in the testing set [23]. A very early prediction model for stroke patients undergoing CABG had an AUC of 0.70 [24]. A multicenter Spanish study established a multivariate prediction model for perioperative in-hospital cerebrovascular accident after coronary bypass surgery, and the AUC was 0.77 [25]. The present study constructed several prediction models using machine learning method, and the Catboost model had the optimum predictive value for the risk of stroke in CAD patients who underwent coronary revascularization. The prediction model can handle irregular data, missing values and other problems well, and has good robustness, and effectively prevent overfitting, which also makes the model more general; in addition, it can match any advanced machine learning algorithm in terms of model performance [20]. The prediction model might provide an easy tool for the clinicians to quickly identify CAD patients undergoing coronary revascularization who were at high risk of stroke. The success of deep learning and machine learning has brought excitement and high expectations in revolutionary changes in health care in CAD patients [26–28]. The deep learning and machine learning algorithm could achieve more accurate results and outperform statistical methods. The findings of this study might be interesting for other researchers from different fields.

CCI is a measure of comorbidity burden that facilitates the evaluation of the prognostic significance of various clinical conditions based on their quantity and individual prognostic impact [29]. CCI has been extensively investigated in various clinical conditions and its significance as a prognostic indicator has been demonstrated. A previous study depicted that CCI was higher in patients with a more diffuse extent of CAD than those with milder disease [30]. Rashid et al. indicated that CCI >2 significantly increased the risk of mortality in acute coronary syndrome [31]. CCI was also identified to be a predictor of readmission in CAD patients [32]. CCI was reported to be highly associated with long-term survival and almost equivalent to left ventricular ejection fraction [33]. CCI was independently associated with an increase in 30-day, 1-year and 5-year cardiac death and major adverse cardiovascular events [34]. Other studies also revealed that CCI was a reliable indicator for the mortality of ischemic stroke patients [35–37]. Herein, CCI was found to be a vital predictor for the risk of stroke in CAD patients who received coronary revascularization. Age was identified to be an important variable associated with the outcomes of post-ST-segment elevation myocardial infarction patients among patients without preexisting coronary artery disease [38]. In this study, age was also related to the risk of stroke in CAD patients who received coronary revascularization.

Several prediction model for stroke risk in CAD patients with coronary revascularization treatments was constructed based on a variety of machine learning methods, which might provide certain references for early identification of high-risk patients and management of postoperative complications. Some limitations existed in this study. Firstly, although all the data were divided into the training set and testing set, the samples were from a single center, and the model should be applied with caution. Secondly, due to the limitations of the MIMIC database, some preoperative and postoperative data, and data related to liver enzymes could not be obtained. More studies were required to verify the results in this study.

## Conclusions

The current study established several prediction models and identified an optimal model for the risk of stroke in CAD patients who received coronary revascularization. The prediction model might offer quick tool to identify CAD patients receiving coronary revascularization who were at high risk of stroke, and make specific treatment strategies to prevent the occurrence of stroke.
